# Prevalence and determinants of postpartum depression among postnatal clinic attendees at the Gwarinpa General Hospital, Abuja, Nigeria

**DOI:** 10.11604/pamj.2026.54.14.49522

**Published:** 2026-05-19

**Authors:** Olubunmi Adeyemi, Fatima Sani, Adedayo Adeyemi, Elvis Efe Isere, Amos Bassi

**Affiliations:** 1 Department of Community Medicine, Nile University of Nigeria, Abuja, Nigeria,; 2 Public Health Department, Federal Capital Territory Administration, Abuja, Nigeria,; 3 Center for Infectious Diseases, Research and Evaluation, Lafia, Nigeria,; 4 School of Public Health, University of Port Harcourt, Rivers State, Nigeria

**Keywords:** Postpartum depression, postnatal care, prolonged labour, maternal mental health, Nigeria

## Abstract

**Introduction:**

postpartum depression (PPD) is a major global public health concern with profound effects on maternal and child wellbeing. It remains one of the most common but under-recognised complications of childbirth, with higher prevalence in low- and middle-income countries, including Nigeria. Limited data are available on the prevalence of PPD in Nigeria, despite unique sociodemographic and psychosocial contexts. This study assessed the prevalence of PPD and its associated sociodemographic, obstetric, and psychosocial factors among pregnant women attending a 6-week postnatal clinic at the Gwarinpa General Hospital, Abuja, Nigeria.

**Methods:**

a cross-sectional study of 400 postpartum women was conducted using systematic random sampling. Data was collected using interviewer-administered questionnaires on sociodemographic, obstetric, and psychosocial factors. The Edinburgh Postnatal Depression Scale (EPDS) was used to assess depressive symptoms (cut-off ≥9). Analysis employed descriptive statistics, chi-square, and logistic regression with significance at p < 0.05.

**Results:**

PPD prevalence was 19.0% (76/400). Most sociodemographic and obstetric factors were not significantly associated with PPD. However, prolonged labour was a strong predictor. Women with labour lasting >12 hours had significantly higher odds of PPD (OR = 3.88, 95% CI: 1.64-9.16).

**Conclusion:**

postpartum depression had a prevalence of nearly one in five women in this study population, and prolonged duration of labour was significantly associated with PPD. These findings highlight the importance of strengthening intrapartum care and integrating targeted postpartum mental health screening for women who experience prolonged labour.

## Introduction

Maternal mental health is a core component of reproductive health and a critical determinant of outcomes for women, newborns, and families [1-3]. Postpartum depression (PPD) is among the most common mental disorders in the postnatal period and represents a significant public health concern in Nigeria [2-4]. Postpartum depression (PPD) is a depressive disorder occurring within weeks to months after childbirth and is characterized by persistent low mood, anhedonia, irritability, sleep and appetite disturbances, impaired mother-infant bonding, and, in severe cases, suicidal ideation or thoughts of harming the infant [4-7].

Globally, PPD affects approximately 10-20% of postpartum women [7-9]. The burden is higher in low- and middle-income countries (LMICs), including Nigeria, where prevalence estimates range from 15% to 57% [8-10]. In Africa, pooled prevalence is estimated at 18.4%, while sub-Saharan Africa reports rates between 16.8% and 18.6% [8,10]. Within Nigeria, regional studies report prevalence rates of 14.6% in the Southwest [11,12], 22.9% and 34.6% in Enugu (Southeast) [13,14], and 21.8% in Jos (North Central) [15], highlighting the substantial and heterogeneous burden across geopolitical zones [8,14,16,17].

Established predictors of PPD include poor social support, marital dissatisfaction, unintended pregnancy, obstetric complications, and socioeconomic adversity [5,18]. Psychosocial stressors such as intimate partner violence, low self-esteem, and ineffective coping mechanisms further increase vulnerability [19]. In Nigeria, sociocultural norms and stigma surrounding mental illness contribute to underreporting, delayed help-seeking, and limited integration of mental health screening into routine postnatal care [8,9,14-17,20]. Consequently, many cases remain undetected despite the profound consequences of untreated PPD, including impaired maternal-infant bonding, suboptimal breastfeeding, adverse child neurodevelopmental outcomes, and increased risk of maternal suicide [7,21].

Although several Nigerian studies have examined PPD, important geographical gaps persist [8,9,14-17]. Most research has been conducted in southern or semi-urban/rural settings, with limited representation of rapidly urbanizing metropolitan areas like Abuja [8,9,14-17,22-24]. Additionally, hospital-based data on PPD are limited, constraining evidence-informed integration of mental health care into postnatal services. Women experiencing obstetric complications may be at higher risk, yet prevalence and determinants within hospital postnatal clinics remain under-investigated. This study provides facility-level evidence, which is essential for designing a contextualized screening model, risk stratification, support services, and targeted interventions in postnatal care service delivery. This study, therefore, assessed the prevalence of PPD and its associated sociodemographic, obstetric, and psychosocial predictors among women attending a 6-week postnatal clinic in Abuja, Nigeria.

## Methods

**Study area and study site:** Gwarinpa General Hospital is a major healthcare facility in Abuja, Federal Capital Territory (FCT), Nigeria [25,26]. Serving Gwarinpa Estate, Jabi, Life-Camp, and nearby suburbs like Karimo, Gwagwa, and the Idu industrial area, its strategic location and standard healthcare service delivery make it a convenient choice for residents seeking medical care [25,26]. The hospital offers a range of services, including maternal and child health, reproductive health, and specialized care [25,26].

**Study population:** the study population consists of the women of reproductive age attending the postnatal clinic of Gwarinpa General Hospital in Abuja, FCT.

### Eligibility criteria

**Inclusion criteria:** participant must be ≥18 years; women of age (18-49 years) who attended the 6-week (42^nd^ day following childbirth) postnatal clinic at Gwarinpa General Hospital, Abuja, FCT; women who had been resident in Abuja, FCT for at least 6 months prior to the index delivery; women who could communicate in English or Hausa (languages used for the interviewer-administered questionnaire).

**Exclusion criteria:** women with a known history of psychiatric or severe mental illness diagnosed before the index pregnancy; mothers with severe medical complications (e.g., eclampsia, postpartum sepsis) that impaired participation at the time of data collection.

**Study design:** this is a descriptive cross-sectional study.

**Sample size determination:** the sample size was determined using Fisher´s formula for a single population proportion. A prevalence (p) of 35.6%, based on previously reported PPD prevalence in Lagos, Nigeria, was used [8]. Assuming a 95% confidence level (Z = 1.96) and 5% margin of error, the minimum sample size calculated was 353 participants. To account for a 10% attrition rate, the sample size was adjusted to 392 participants.

**Sampling technique:** a systematic random sampling technique was employed. The postnatal care (PNC) clinic is held for four days per week (Monday to Thursday) with average monthly attendance rates of 840. Data was collected over a period of four weeks (16 antenatal clinic (ANC) days). The sampling interval calculated as = 840/392 = 2.1 ≈ 3. The number of pregnant women pre-determined to be sampled per day was 25 (392/16). Therefore, with a sampling interval of 3, the first to be sampled per clinic day was selected from the first 3 persons by a simple random sampling, after which every third person was selected until 25 persons were selected per clinic day by three (3) trained research assistants in four weeks.

**Postpartum depression diagnosis criteria:** postpartum depression among study participants was assessed using the Edinburgh Postnatal Depression Scale (EPDS), a widely used and validated screening instrument for detecting depressive symptoms during pregnancy and the postpartum period [27,28]. At each woman´s follow-up visit to the PNC clinic on the 42^nd^ day postpartum, participants were evaluated for a range of depressive symptoms, including a reduced ability to laugh and see the funny side of things, diminished enjoyment or anticipation of daily activities, feelings of unnecessary self-blame when things went wrong, anxiety or excessive worry without an apparent reason, episodes of fear or panic, a sense of being overwhelmed or unable to cope with everyday demands, sleep disturbances related to unhappiness, persistent sadness or feelings of misery, frequent crying due to emotional distress, and thoughts of self-harm as described by Cox *et al*. [27] in the original development of the EPDS.

The EPDS is a self-report tool consisting of 10 items, each scored on a 4-point scale ranging from 0 to 3, reflecting the respondent´s experiences over the previous seven days. The total possible score ranges from 0 to 30, with higher scores indicating greater severity of depressive symptoms [27-29]. The scale has been extensively validated for use during both pregnancy and the postpartum period across diverse populations [27-33].

For the purpose of this study, an EPDS score of ≥9 was used as the diagnostic criterion to identify the presence of postpartum depressive symptoms, while respondents with scores <9 were classified as not having postpartum depression [29]. This cut-off point was defined prior to data collection and analysis and served as the basis for identifying PPD cases among the study participants.

Although an EPDS score of ≥13 is commonly used in clinical settings to identify women at risk of major depressive disorder, a lower threshold of ≥9 was applied in this study to enhance the sensitivity for detecting depressive symptoms within the study population [29,34]. The choice of this threshold was informed by evidence indicating that the optimal EPDS cut-off may vary across cultural and socioeconomic contexts, particularly in LMICs where mental health conditions are often underdiagnosed due to stigma, limited access to care, and low levels of mental health awareness [29,34].

Studies conducted in similar settings have demonstrated that a lower cut-off point, such as ≥9, improves sensitivity for identifying a broader spectrum of depressive symptoms, including mild to moderate depression, which remain clinically significant and warrant attention [29,34]. This classification was subsequently used to define the binary outcome variable (PPD: yes/no) for estimating the prevalence of postpartum depression and examining its association with selected sociodemographic, obstetric, and psychosocial factors.

**Data collection:** data was collected over a period of four (4) weeks by the investigators and three (3) trained research assistants who are fluent in both English and Hausa languages. The research data collection tool for this study was adapted from a study conducted in a comparable Nigerian context [8] to ensure cultural relevance and methodological appropriateness. Additionally, the tool was pretested at Maitama District Hospital to evaluate its clarity, reliability, and suitability for the local population, allowing necessary adjustments before the main study. The finalized data collection tool consisted of a structured interviewer-administered questionnaire containing different sections, which include section A, socio-demographic characteristics of respondents, with sections B, C, and D capturing information on birth experiences, PPD, and factors associated with PPD, respectively.

**Data analysis:** data collected from respondents were entered, cleaned, and analysed using IBM Statistical Package for Service Solution (SPSS) version 25. Prior to analysis, the dataset was checked for completeness, internal consistency, and possible entry errors. Descriptive statistics were used to summarise the socio-demographics, obstetrics, and other related characteristics of the study population. Continuous variables were summarised using means and standard deviations, while categorical variables were presented as frequencies and percentages in tables.

**Estimation of the prevalence of postpartum depression:** to calculate the prevalence of postpartum depression, the number of respondents who screened positive for PPD (EPDS score ≥9) was divided by the total number of postnatal women included in the study and multiplied by 100. Thus, the prevalence of PPD was calculated as: prevalence of PPD = (number of respondents with EPDS score ≥ 9 ÷ total number of respondents assessed) x 100.

**Factors associated with postpartum depression:** the association between postpartum depression and selected sociodemographic, obstetric, and psychosocial characteristics was assessed using the Pearson chi-square test. Variables examined included age, marital status, educational attainment, occupation, income level, social support, gravidity, parity, and duration of labour.

Variables that were statistically significant (p < 0.05) in the bivariate analysis were further included in a binary logistic regression model to identify independent predictors of postpartum depression. The strength of association between explanatory variables and PPD was expressed using odds ratios (ORs) with corresponding 95% confidence intervals (CIs). A p-value of less than 0.05 was considered statistically significant for all analyses.

**Ethical approval and consent to participate:** this study was conducted in accordance with the ethical principles outlined in the Declaration of Helsinki. Ethical approval was obtained from the University of Jos Teaching Hospital Research Ethics Committee, Nigeria, with protocol number: FHREC/2022/01/59/07-04-22. Also, informed consent was obtained from the respondents. Before data collection, respondents were approached individually by trained research assistants and given a detailed explanation of the study. The research assistants introduced themselves, explained the study´s objectives, and ensured that each respondent understood the purpose of their participation. The explanation was provided in a language (English, Hausa, or Pidgin) that the respondents were comfortable with to enhance comprehension. Respondents were informed that participation was entirely voluntary, meaning they had the right to refuse or withdraw at any time without facing any consequences. They were reassured that their decision would not affect their access to healthcare services or other benefits of the study.

To formalize consent, respondents were provided with an informed consent form outlining the study´s purpose, procedures, potential risks, benefits, and confidentiality measures. If literate, they were asked to read and sign the form. If illiterate, a witness who could read and write was asked to interpret the content of the form and explain it to the understanding of the respondent, after which the respondent provided consent through a thumbprint, witnessed by the interpreter. Signed or thumbprinted forms were securely stored as proof of consent before proceeding with data collection. Data collected from respondents were kept confidential using a password-secured ODK database with access provided only to the principal investigator.

## Results

**Sociodemographic characteristics of respondents:** among the 400 respondents surveyed, more than one third were aged 26-30 years (164 (41.0%)), and while most respondents were married (388 (97.0%)). Most marriages were monogamous (345 (88.9%) of 388 married women). The dominant ethnic groups were Igbo (144 [36.0%]) and Yoruba (98 (24.5%)). Christianity was the most practiced religion (278 (69.5%)).

Over half of the respondents had attained tertiary education (212 (53.0%)), and above one third had secondary education (147 (36.8%)). Most women reported small family sizes, with 284 (71.0%) having two children. About one third of the respondents, 132 (33.0%), were unskilled, while 99 (24.8%) held non-manually skilled jobs. About three-quarters of the respondents, 302 (75.5%), reported tertiary educational attainment among their spouses (Table 1). The predominant income bracket was ₦ 50,000-99,000 per month (291 (72.8%)).

**Table 1:** sociodemographic characteristics of postnatal women attending the 6-week postnatal clinic at the Gwarinpa General Hospital, Abuja, Nigeria (n = 400)

Variable	Frequency (n = 400)	Percent
**Age (year)**		
18-20	16	4.0
21-25	45	11.3
26-30	164	41.0
31-35	86	21.5
≥40	89	22.3
**Marital status**		
Married	388	97.0
Not married	12	3.0
Marriage type, n = 388		
Monogamy	345	88.9
Polygamy	43	11.1
**Ethnicity**		
Hausa/Fulani	97	24.3
Igbo	144	36.0
Yoruba	98	24.5
Others*	61	15.3
**Religion**		
Christianity	278	69.5
Islam	122	30.5
**Family size**		
2	284	71.0
≥3	116	29.1
**Education**		
No formal	16	4.0
Primary	25	6.3
Secondary	147	36.8
Tertiary	212	53.0
**Occupation**		
Unskilled	132	33.0
Semi-skilled	49	12.3
Manually skilled (Artisan)	76	19.0
Non-manually skilled	99	24.8
Intermediate/Professional	44	11
**Estimated monthly income (₦)**		
<50,000	11	2.8
50,000-99,000	291	72.8
≥100,000	98	24.6

**Prevalence of PPD among respondents:** the mean EPDS score among respondents was 6.9 ± 2.0. Using the predefined cut-off score, 76 (19.0%) respondents screened positive for postpartum depression, while 324 (81.0%) had scores below the diagnostic threshold. Notably, over one-third (35.3%) of respondents who experienced labour lasting more than 12 hours reported symptoms of PPD, compared to 12.3% among those whose labour lasted less than 6 hours (Table 2).

**Table 2:** prevalence of postpartum depression among postnatal women attending the 6-week postnatal clinic at the Gwarinpa General Hospital, Abuja, Nigeria (n = 400)

Variable	Frequency (n = 400)	Percentage (%)
**Last pregnancy planned**		
Yes	281	70.3
No	119	29.7
**Sex of last baby**		
Male	198	49.5
Female	202	50.5
**Sex of last baby is the mother wanted**		
Yes	176	44.0
No	224	56.0
**Have any social support**		
Yes	297	74.3
No	103	25.7
**Experienced any form of violence**		
Yes	5	1.2
No	395	98.8
**Family history of depression**		
Yes	2	0.5
No	398	99.5
**History of hormonal imbalance**		
Yes	4	1.0
No	396	99.0
**Addiction to alcohol**		
Yes	1	0.2
No	399	99.8
**Take recreational drugs**		
Yes	3	0.7
No	397	99.3
**Personality**		
Introvert	119	29.8
Perfectionist	81	20.3
Sensitive to criticism	110	27.5
Someone with low self-esteem	90	22.5
**Presence of chronic illness**		
Yes	53	13.2
No	347	86.8
**Chronic illness, n = 53**		
Asthma	17	32.1
Diabetes	25	47.2
Hypertension	11	20.7
**Experienced life event**		
Yes	5	1.2
No	395	98.8
**Life event experienced, n = 5**		
Dysfunctional relationships	2	40.0
Grieve	3	60.0

**Maternal obstetric history among respondents:** Table 3 shows the obstetric history of the study participants. More than half of our respondents had experienced two pregnancies (222; 55.5%) and two births (229; 57.3%), respectively, with a low history of child death (14; 3.5%). Similarly, more than half of our respondents had two living children (229; 57.3%) and reported no complications or difficulties during their last pregnancy (393; 98.3%). Most deliveries occurred in health facilities (381; 95.3%) and were attended by skilled birth attendants (397; 99.3%). Vaginal delivery was the most common mode (299; 74.8%), and labour mostly lasted between 6-12 hours (220; 55.0%). Less than one-tenth reported birth complications (9; 2.2%) during the last pregnancy or reported ever having an abortion (27; 6.8%), of which nearly all were spontaneous (26; 96.3%).

**Table 3:** maternal obstetric history of postnatal women attending the 6-week postnatal clinic at the Gwarinpa General Hospital, Abuja, Nigeria (n = 400)

Variable	Frequency (n = 400)	Percent
**Gravidity**		
1	67	16.8
2	222	55.5
≥3	111	27.6
**Parity**		
1	69	17.3
2	229	57.3
≥3	102	25.5
**History of child death**		
Yes	14	3.5
No	386	96.5
**Number of living children**		
1	69	17.3
2	229	57.3
≥3	102	25.5
**Difficulty during last pregnancy**		
Yes	7	1.8
No	393	98.3
**Place of delivery of last pregnancy**		
Health facility	381	95.3
Home	14	3.5
Mission centre	5	1.3
**Delivery by Skilled birth attendant**		
Yes	397	99.3
No	3	0.7
**Mode of delivery**		
Simple vaginal delivery	299	74.8
Caesarean section	101	25.2
**Duration of labour (hour)**		
<6	146	36.5
6-12	220	55.0
>12	34	8.5
**Complication**		
Yes	9	2.2
No	391	97.8
**Ever had an abortion**		
Yes	27	6.8
No	373	93.3
**Type of abortion, n = 27**		
Spontaneous	26	96.3
Induced	1	3.7

**Psychosocial experiences reported by respondents:** Table 4 shows the psychosocial experiences of respondents. More than one-third of the respondents reported that their pregnancies were planned (281; 70.3%), and almost three-quarters of the respondents reported having social support (297; 74.3%). While the sex of babies was nearly equally distributed, over half (224; 56.0%) did not have their preferred sex. Less than one-tenth of the respondents reported experiences of violence (5 (1.2%)), family history of depression (2 (0.5%)), hormonal imbalance (4 (1.0%)), alcohol addiction (1 (0.2%)), or recreational drug use (3 (0.7%)). However, common personality traits reported by the respondents were introversion (119 (29.8%)) and sensitivity to criticism (110 (27.5%)). Chronic illness was reported by 53 (13.2%), mainly diabetes (25 (47.2%)) and asthma (17 (32.1%)). Only 5 (1.2%) had experienced major life events such as grief or dysfunctional relationships.

**Table 4:** psychosocial characteristics of postnatal women attending the 6-week postnatal clinic at the Gwarinpa General Hospital, Abuja, Nigeria (n = 400)

Variable	Frequency (n = 400)	Percent
**Able to laugh and see the funny side of things**		
As much as I always could	112	28.0
Not quite so much now	180	45.0
Not so much now	95	23.8
Not at all	13	3.3
**Looked forward to things with enjoyment**		
As much as I ever did	101	25.3
Rather less than I used to	179	44.8
Less than I used to	89	22.3
Hardly at all	31	7.8
**Blamed oneself unnecessarily when things went wrong**		
No, never	101	25.3
Not very often	275	68.8
Yes, some of the time	15	3.8
Yes, most of the time	9	2.3
**Anxious or worried for no very good reason**		
No, not at all	280	70.0
Hardly ever	108	27.0
Yes, sometimes	7	1.8
Yes, very often	5	1.3
**Was scared or panicky for no very good reason**		
No, not at all	112	28.0
No, not much	269	67.3
Yes, sometimes	13	3.3
Yes, quite a lot	6	1.5
**Things have been getting on top**		
No, I have been coping as well as ever	123	30.8
No, most of the time I have coped quite well	255	63.7
Yes, sometimes I haven't been coping as usual	10	2.5
Yes, most of the time haven't been able to cope at all	12	3.0
**Been so unhappy that have had difficulty sleeping**		
No, not at all	40	10.0
Not, very often	281	70.3
Yes, sometimes	49	12.3
Yes, most of the time	30	7.5
**Have felt sad or miserable**		
No, not at all	250	62.5
Not, very often	130	32.5
Yes, sometimes	14	3.5
Yes, most of the time	6	1.5
**Have been so unhappy they have been crying**		
No never	350	87.5
Only occasionally	40	10.0
Yes, quite often	10	2.5
**Thoughts of harming self has occurred**		
Never	317	79.3
Hardly ever	81	20.3
Sometimes	2	0.5
**+Postpartum depression score (PPD)**		
<9	324	81.0
≥9	76	19.0

+: postpartum depression (PPD) ≥ 9

**Sociodemographic, obstetric and psychosocial factors associated with PPD among respondents:** Table 5 shows household, obstetrics and psychosocial factors associated with PPD among respondents. Among the 400 postnatal women sampled, the PPD prevalence was highest in the 15-25 years age group (23.0%) and lowest in the ≥36 years group (14.6%) (χ^2^= 5.252, p = 0.385). Regarding the marital status of the study participants, unmarried women had a higher PPD prevalence (33.3%) compared to married women (18.6%) (χ^2^= 1.651, p = 0.199). Similarly, by marriage type, women in monogamous relationships recorded a higher PPD prevalence (19.7%) than those who were polygamous (9.2%) (χ^2^= 2.740, p = 0.098). Across religions, the prevalence remained comparable, with Christians at 20.4% and Muslims at 18.9% (χ^2^= 0.002, p = 0.960).

**Table 5:** sociodemographic, obstetric, and psychosocial factors associated with postpartum depression among postnatal women at the 6-week postnatal clinic, Gwarinpa General Hospital, Abuja (n = 400)

Variable	PPD yes n (%)	PPD no n (%)	Total	χ^2^	p-value
**Age (years)**					
15-25	14 (23.0)	47 (77.0)	61	5.252	0.385
26-35	49 (19.6)	201 (80.4)	250		
≥36	13 (14.6)	76 (85.4)	89		
**Marital status**					
Married	72 (18.6)	316 (81.4)	388	1.651	0.199
Not married	4 (33.3)	8 (66.7)	12		
**Marriage type**					
Monogamy	68 (19.7)	277 (80.3)	345	2.74	0.098
Polygamy	8 (14.5)	47 (85.5)	55		
**Ethnicity**					
Hausa/Fulani	20 (20.6)	77 (79.4)	97	1.012	0.798
Igbo	27 (18.8)	117 (81.3)	144		
Yoruba	20 (20.4)	78 (79.6)	98		
Others	9 (14.8)	52 (85.2)	61		
**Religion**					
Christianity	53 (20.4)	226 (79.6)	279	0.002	0.96
Islam	23 (18.9)	99 (81.1)	122		
**Education**					
No formal	4 (25.0)	12 (75.0)	16	4.354	0.226
Primary	4 (16.0)	21 (84.0)	25		
Secondary	35 (23.6)	112 (76.4)	147		
Tertiary	33 (15.6)	179 (84.4)	212		
**Occupation**					
Unskilled	31 (23.5)	101 (76.5)	132	4.655	0.325
Semi-skilled	11 (22.4)	38 (77.6)	49		
Artisan	10 (13.2)	66 (86.8)	76		
Non-manual	18 (18.2)	81 (81.8)	99		
Professional	6 (13.6)	38 (86.4)	44		
**Income (₦)**					
<50,000	1 (9.1)	10 (90.9)	11	1.481	0.687
50k-99k	55 (18.9)	236 (81.1)	291		
≥100k	20 (20.4)	78 (79.6)	98		
**Social support**					
Yes	51 (17.2)	246 (82.8)	297	2.505	0.113
No	25 (24.3)	78 (75.7)	103		
**Gravidity**					
1	17 (25.4)	50 (74.6)	67	2.748	0.258
2	42 (18.9)	180 (81.1)	222		
≥3	17 (15.3)	94 (84.7)	111		
**Parity**					
1	17 (24.6)	52 (75.4)	69	2.654	0.265
2	44 (19.2)	185 (80.8)	229		
≥3	15 (14.7)	87 (85.3)	102		
**Sex of baby**					
Male	42 (21.2)	156 (78.8)	198	1.247	0.264
Female	34 (16.8)	168 (83.2)	202		
**Violence experienced**					
Yes	1 (20.0)	4 (80.0)	5	0.003	0.954
No	75 (19.0)	320 (81.0)	395		
**Complication during pregnancy**					
Yes	2 (22.2)	7 (77.8)	9	0.062	0.803
No	74 (18.9)	317 (81.1)	391		
**Duration of labour**					
<6 hrs	18 (12.3)	128 (87.7)	146	10.609	0.005*
6-12 hrs	46 (20.9)	174 (79.1)	220		
>12 hrs	12 (35.3)	22 (64.7)	34		

* : statistically significant

Additionally, among the 400 study participants, PPD prevalence was highest among those with no formal education (25.0%) and lowest among those with tertiary education (15.6%) (χ^2^= 4.354, p = 0.226). Unskilled workers had the highest PPD prevalence (23.5%), while artisans had the lowest (13.2%) (χ^2^= 4.655, p = 0.325). PPD was higher among women without social support (24.3%) than those with support (17.2%) (χ^2^= 2.505, p = 0.113). Notably, the duration of labour showed a statistically significant association (χ^2^= 10.609, p = 0.005). PPD prevalence increased with labour length, from 12.3% among women with labour lasting less than 6 hours to 35.3% among those with labour exceeding 12 hours.

**Association between duration of labour and PPD:** Table 6 presented the results of a logistic regression analysis examining the association between duration of labour and the likelihood of PPD, with labour lasting less than 6 hours serving as the reference category. Compared with this group, respondents who experienced labour lasting 6-12 hours had significantly higher odds of PPD (unadjusted odds ratio (OR) = 1.88, 95% CI: 1.04-3.39, p = 0.036). Respondents whose labour exceeded 12 hours had higher odds of PPD (unadjusted OR = 3.88, 95% CI: 1.64-9.16, p = 0.002).

**Table 6:** association between duration of labour and postpartum depression among postnatal women at the 6-week postnatal clinic, Gwarinpa General Hospital, Abuja (n = 400)

Variables	Unadjusted Odds Ratio (95% CI)	p-value
**Duration of labor (hours)**		
< 6	R	
6-12	1.88 (1.04-3.39)	0.036*
> 12	3.88 (1.64-9.16)	0.002*

* : statistically significant; OR: odds ratio

## Discussion

The findings of this study provide compelling evidence for a significant association between duration of labour and the development of PPD. Specifically, the results reveal a dose-response relationship in which women with labour durations exceeding 12 hours had almost four times the odds of experiencing PPD compared to those with shorter durations. This observed prevalence of 19% aligns with global estimates ranging from 10% to 20% [35,36], yet it stands in stark contrast to the 5.7% reported in Kaduna State by Mohammed-Durosinlorun *et al*. [37]. This difference may be proffered by the specific demographic profile of our participants; our study involved a high percentage of women with tertiary education (53%) and professional occupations, who may possess higher health literacy and a greater readiness to acknowledge and report depressive symptoms compared to more conservative or rural cohorts where mental health stigma remains a significant barrier to disclosure [11].

The relationship between prolonged labour and increased PPD risk is likely rooted in both physiological and psychological mechanisms. Physiologically, the exhaustion of a labour lasting over 12 hours can lead to a sustained dysregulation of the Hypothalamic-Pituitary-Adrenal (HPA) axis and a protracted stress response, which are established biological precursors to depression [38-42]. Psychologically, our findings support the research of Kurth *et al*. [43] and Garthus-Niegel *et al*. [44], suggesting that the “traumatic” element of a long labour characterized by a loss of control and fear for fetal safety functions as a primary psychological stressor. This is particularly relevant in our study, as 56% of mothers reported that the sex of the baby was not what they had originally wanted, which, when coupled with a difficult delivery, may compound feelings of disappointment and maternal inadequacy.

Interestingly, several variables typically associated with PPD, such as social support and mode of delivery, were not statistically significant in this study [42-44]. While lack of social support is a widely documented risk factor [45], the high prevalence of reported support in our sample (74.3%) likely created a “ceiling effect,” where the relative abundance of family assistance across both groups obscured the impact of social isolation found in other studies. Furthermore, the non-significance of the mode of delivery (caesarean section vs. spontaneous vaginal delivery) differs from some global literature but may be explained by the high rate of skilled birth attendance (99.3%) and facility-based deliveries (95.3%) among our respondents [46]. In this context, the quality of clinical care and the presence of professional support may have mitigated the psychological distress often associated with surgical interventions [47]. Additionally, marital status and marriage type were not significant predictors of PPD in this study, a finding likely influenced by the low variability in our sample, as 97% of respondents were married and 88.9% were in monogamous unions. Unlike studies in populations with higher rates of single parenthood or polygamy, where economic instability and lack of partner intimacy are major drivers of PPD [48], our study participants appear to be relatively stable in terms of household structure. This suggests that in this specific population, the homogeneity of social standing may have masked the impact of marital stressors found in more diverse cohorts [49]. Consequently, obstetric factors, namely the physical and temporal experience of birth, emerge as more dominant predictors of PPD than traditional sociodemographic stressors. Overall, these findings underscore the importance of context, measurement tools, and sample variability when assessing risk factors for PPD and suggest the need for future research.

Nevertheless, the study is not without limitations. The cross-sectional design limits the study to descriptive and associative interpretations. Furthermore, the reliance on self-reported depressive symptoms, although based on a validated screening tool, may be subject to reporting bias or cultural influences on emotional disclosure. Additionally, social desirability bias might have occurred in some instances because respondents may respond to interview questions in a way that they believe is socially acceptable rather than being completely accurate. However, probing questions were asked to ensure correct responses where possible.

## Conclusion

Postpartum depression had a prevalence of nearly one in five women in this study population, and prolonged duration of labour was significantly associated with PPD. These findings highlight the importance of strengthening intrapartum care and integrating targeted postpartum mental health screening for women who experience prolonged labour. Further longitudinal and interventional studies are warranted to explore the causal pathways and develop targeted preventive strategies.

### What is known about this topic

- Postpartum depression is a common but under-recognised complication of childbirth globally, with higher prevalence in low- and middle-income countries, including Nigeria;
- Multiple sociodemographic, obstetric, and psychosocial factors such as poverty, poor social support, unplanned pregnancy, and delivery complications are established risk factors for PPD.

### What this study adds

- Identified prolonged labour (>12 hours) as an independent risk factor for PPD, emphasizing the significance of obstetric complications in informing targeted maternal mental health screening and postnatal care interventions.
